# Photobiomodulation Protects and Promotes Differentiation of C2C12 Myoblast Cells Exposed to Snake Venom

**DOI:** 10.1371/journal.pone.0152890

**Published:** 2016-04-08

**Authors:** Luciana Miato Gonçalves Silva, Camila Aparecida Alves da Silva, Aline da Silva, Rodolfo Paula Vieira, Raquel Agnelli Mesquita-Ferrari, José Carlos Cogo, Stella Regina Zamuner

**Affiliations:** 1 Posgraduated Program in Medicine, Universidade Nove de Julho–UNINOVE, São Paulo, SP, Brazil; 2 Posgraduated Program in Biophotonics, Universidade Nove de Julho–UNINOVE, São Paulo, SP, Brazil; 3 Institute of Research and Development, Universidade do Vale do Paraíba, UNIVAP, São José dos Campos, SP, Brazil; University of Minnesota Medical School, UNITED STATES

## Abstract

**Background:**

Snakebites is a neglected disease and in Brazil is considered a serious health problem, with the majority of the snakebites caused by the genus *Bothrops*. Antivenom therapy and other first-aid treatments do not reverse local myonecrose which is the main sequel caused by the envenomation. Several studies have shown the effectiveness of low level laser (LLL) therapy in reducing local myonecrosis induced by Bothropic venoms, however the mechanism involved in this effect is unknown. In this *in vitro* study, we aimed to analyze the effect of LLL irradiation against cytotoxicity induced by *Bothrops jararacussu* venom on myoblast C2C12 cells.

**Methodology:**

C2C12 were utilized as a model target and were incubated with *B*. *jararacussu* venom (12.5 μg/mL) and immediately irradiated with LLL at wavelength of red 685 nm or infrared 830 nm with energy density of 2.0, 4.6 and 7.0 J/cm^2^. Effects of LLL on cellular responses of venom-induced cytotoxicity were examined, including cell viability, measurement of cell damage and intra and extracellular ATP levels, expression of myogenic regulatory factors, as well as cellular differentiation.

**Results:**

In non-irradiated cells, the venom caused a decrease in cell viability and a massive release of LDH and CK levels indicating myonecrosis. Infrared and red laser at all energy densities were able to considerably decrease venom-induced cytotoxicity. Laser irradiation induced myoblasts to differentiate into myotubes and this effect was accompanied by up regulation of MyoD and specially myogenin. Moreover, LLL was able to reduce the extracellular while increased the intracellular ATP content after venom exposure. In addition, no difference in the intensity of cytotoxicity was shown by non-irradiated and irradiated venom.

**Conclusion:**

LLL irradiation caused a protective effect on C2C12 cells against the cytotoxicity caused by *B*. *jararacussu* venom and promotes differentiation of these cells by up regulation of myogenic factors. A modulatory effect of ATP synthesis may be suggested as a possible mechanism mediating cytoprotection observed under laser irradiation.

## Introduction

Local acute skeletal muscle injury is a common manifestation caused by envenomation from snakes of Bothrops genus that leads to necrosis with consequent loss of muscle mass, which represents the main sequel of this envenoming [1–4]. *Bothrops jararacussu (B*. *jararacussu)* is the main venomous snake in southeast region of Brazil and northern Argentina, and its venom presents strong myotoxic effect [5]. The miotoxic effect caused by *B*. *jararacussu* venom is due to a large amount of myotoxins present in this venom, which damage the plasma membrane of muscle cells, causing myonecrosis [6].

The parenteral administration of antivenoms constitutes the mainstay in the therapy of snakebite envenoming [7]. This therapy is efficient to minimize the systemic effects when administered rapidly after the bite and may prevent death. In contrast, antivenom therapy does not prevent local tissue damage leading to a functional or even anatomical loss of the affected limb with important physical, psychological and social consequences [6, 8, 9]. Thus, alternative therapies to prevent or even counteract this serious local consequence of snakebite envenomation are of great importance.

Photobiomodulation is a form of light therapy that utilizes non-thermal irradiation forms of light, including low level laser (LLL) and light emitting diode (LED) within the red or infrared range of light spectrum. Light therapy has been shown, both in experimental model and medical applications, to induce biological activities such as cellular migration and proliferation of many cell types, augmenting tissue repair and regeneration of different tissues and reduction of pain and inflammation [10–15]. The mechanism associated with the cellular photobiomodulation is not yet fully understood. However, the classical mechanism involved in the stimulatory effect of photobiomodulation is based on light absorption by intracellular chromophores located within the mitochondria and converted to metabolic energy leading to adenosine triphosphate (ATP) production, resulting at the end in diverse intracellular signaling pathways activation [16, 17].

In recent years, several experimental studies from our and other groups have described the potential capacity of photobiomodulation to reduce local effects caused by Bothrops venoms. Myonecrosis [18], local inflammation (edema and leukocyte influx) [19, 20], hyperalgesia [20] and blocking of neuromuscular transmission caused by *B*. *jararacussu* venom has been shown to be reduced after LLL irradiation [21]. Moreover, it has been demonstrated that application of Ga-As laser and LED irradiation reduces the local effects induced by *Bothrops moojeni* venom and the authors suggested that the photobiomodulation effect is due to phagocytosis stimulation, myoblasts proliferation and regeneration of muscle fibers [22–25]. In addition, vascular endothelial growth factor receptor-1 (VEGFR-1) expression, and its modulation by HeNe or GaAs laser, has been demonstrated in endothelial and non-endothelial cells of snake envenomed skeletal muscle [26]. We also demonstrated that laser irradiation reduced local effect of isolated snake myotoxins on the inflammatory response and myonecrosis when injected in mice [27, 28]. Although several studies have demonstrated the effectiveness of photobiomodulation in reducing local effects induced by bothrops venom, especially myonecrosis, the mechanism involved in this protection is unknown.

The use of laser irradiation might be a promising approach to improve muscle recovery and healing process after snakebite accidents. Furthermore, the establishment of an effective therapeutic resource to minimize the local venom-induced local effects is essential. Therefore, in the current study we aimed to investigate the ability of laser irradiation to protect C2C12 cells against *B*. *jararacussu* venom. Moreover, we evaluated whether the photobiomodulation affects the capability of C2C12 myoblasts to differentiate after venom incubation.

## Methods

### *B*. *jararacussu* venom

*B*. *jararacussu* venom was supplied from the Center of Studies of the Nature at UNIVAP. The venom was lyophilized and kept under refrigeration at 4°C, being diluted in culture medium immediately before use.

### Cell Culture

The murine C2C12 cell line (ATCC) was used as the venom target. For maintenance of C2C12 myoblast, cells were cultured in growth medium consisting of Dulbeccos modified Eagles medium (DMEM, Cultilab, Campinas, SP, Brazil) supplemented with heat-inactivated 10% fetal bovine serum and 1% antibiotic-antimycotic solution in a humidified atmosphere of 5% CO_2_ at 37°C. Growth medium was changed every two days.

### Experimental groups

Cells were divided into four experimental groups: (1) Control (cells non-irradiated); (2) Venom (cells incubated with *B*. *jararacussu* venom); (3) venom + 685 nm (cells incubated with venom and immediately irradiate with laser at 685 nm); (4) venom + 830 nm (cells incubated with venom and immediately irradiate with laser at 830 nm). As there are no defined parameters in the literature for experiments involving low-level laser and C2C12 myoblast cells exposed to venom, the experimental groups were irradiated with two wavelengths (685 nm and 830 nm) at different energy densities 2.0, 4.6 and 7.0 J/cm^2^. The venom dose used was 12.5 μg/mL and was chosen on the basis of previous study from our group which showed that a dose of 12.5 μg/mL decrease 50–60% cell viability in the period of 15 to 60 min [29].

### Laser irradiation

A low-level semiconductor Ga-As laser, Theralase D.M.C. (São Carlos, SP, Brazil), operating with a wavelength of red 685 nm or infrared 830 nm, was used through the whole experiment to irradiate the cells with a beam spot of 0,28 cm^2^. The output power was 35 mW for the 685 nm and 100 mW for the 830 nm wavelength. The energy density and exposure time are displayed in Table 1. The laser doses, low enough to avoid any thermal effect, was chosen on the basis of studies reported in the literature that had shown a beneficial effect of the low-level laser in cultured cells [30]. Cells were irradiated immediately upon addition of the venom in the culture and were applied directly into the well from the bottom plate in a continuous mode. The experiments were conducted in an environment with partial obscurity to not suffer interference from external light. The output power of the laser equipment was measured using the Laser Check® power meter (MM Optics, São Carlos, Brazil).

**Table 1:** Laser parameter setting.

Wavelengt (nm)	Power (mW)	Exposure Duration (s)	Energy Density (J/cm^2^)
**685**	35	16	2.0
**685**	35	37	4.6
**685**	35	56	7.0
**830**	100	6	2.0
**830**	100	13	4.6
**830**	100	20	7.0

### Irradiated venom

To verify if the laser irradiation could change the venom toxicity, the lyophilized venom of *B*. *jararacussu* was diluted in medium solution and irradiated immediately before the incubation with C2C12 cells, using the same laser parameters used to irradiate the cells. The purpose of this experiment was to clarify if the photobiomodulation can modify the biological activity of the venom.

### Cell viability

The C2C12 cells (1 x10^4^ cells) which had been cultured in 96-well plates under optimal conditions near to total confluence were irradiated and incubated with venom for 15, 30 and 60 min, according to experimental groups. At the fixed time intervals viability was performed by a 3-[4,5-Dimethylthiazol-2yl]-2,5-diphenyltetrazolium bromide (MTT) colorimetric assay (Sigma Aldrich, St. Louis, MO, USA). In brief, the cells were incubated with MTT dye solution to a final concentration of 0.5 μg/ml for 3 h. The medium was removed and the amount of reduced MTT dye was solubilized by isopropanol and measured using an ELISA reader at 620 nm. This assay measures the activity of living cells via mitochondrial dehydrogenase activity that reduces MTT to purple formazan.

### Cytotoxicity assay

The integrity of cell membrane was assessed by determining the release of the cytosolic enzyme lactic dehydrogenase (LDH). Briefly, myoblast cultures (1 x10^4^ cells) in a 96 well plate were incubated with *B*. *jararacussu* venom and laser irradiated according to experimental groups. Aliquots of the supernatant in culture wells were collected at the indicated time intervals, and LDH activity was determined by using a commercial kit (Labtest, Minas Gerais, Brazil). Reference controls for 0% and 100% cytolysis consisted of medium alone and medium from cells incubated with 0.1% (v/v) of Triton X-100, respectively. The myotoxic effect of the venom was additionally evaluated by measuring creatine kinase (CK) activity using a commercial kit CK NAC (Larborlab, São Paulo, Brazil). CK activity was determined as an estimator of necrosis. Values of CK activity were expressed in U/L, one unit resulting in the phosphorylation of one nanomole of creatine per min at 25°C. Each sample was assayed in triplicate wells, in at least three independent experiments.

### Differentiation of C2C12 myoblast

To verify whether the laser would be capable of causing differentiation and myotube formation after incubation with the venom, the morphology of C2C12 cells was assessed after 4 days of myogenic differentiation induction. In brief, the cells, grown with the containing 10% fetal bovine serum medium and attained 80% cell confluence, were analyzed and considered undifferentiated cells. Undifferentiated cells were divided into tubes (1x10^4^ cells), received venom or medium (control) and centrifuged for the formation of cell pellet. They were then irradiated in a punctual manner at the bottom of the tube. Subsequently, the cells were placed on 13 mm cover slides in 24 well plates and incubated for 15, 30 and 60 minutes. To induce myogenic differentiation, cells were switched to DMEM medium containing 2% horse serum to initiate the differentiation and were incubated for 4 days. The myoblast differentiation was assessed by morphological analysis of myotube formation [31].

### Western Blot protein expression

The presence of MyoD and myogenin proteins in C2C12 cell after venom incubation and laser irradiation was detected by Western blotting. Briefly, C2C12 cells were divided into tubes (1x10^4^ cells), received venom or medium (control) and centrifuged for the formation of cell pellet. They were then irradiated in a punctual manner at the bottom of the tube. Subsequently, the cells were placed on 24 well plates and incubated for 15, 30 and 60 minutes. After 3 days, cells were washed with cold phosphate buffer saline (PBS) and directly lysed with Laemmli sample buffer. Then, the samples were run on 10% SDS-PAGE, followed by transfer to a nitrocellulose membrane (Bio-Rad–USA). The membrane was blocked for 1 h at room temperature with 5% non-fat milk, 0.05% Tween-20 in phosphate buffer saline. Then the membrane was incubated with antibodies anti-MyoD (1:500, Abcam®, USA), anti-Myogenin (1:2000, Abcam®, USA), or anti-β-actin, (1:1000, Santa Cruz Biotechnology Inc.) overnight at 4°C. The membrane was washed and incubated with horseradish-peroxidase-conjugated secondary antibody at room temperature for 2 h and the immunoreactive bands were visualized using a chemiluminiscense detection system (Imuno-Star, Bio-Rad). Relative band intensity was quantified using ImageJ software after normalizing with β-actin band intensity.

### Immunohistochemistry

C2C12 cells were divided into tubes (1x10^3^ cells), received venom or medium (control) and centrifuged for the formation of cell pellet. They were then irradiated in a punctual manner at the bottom of the tube. Subsequently, C2C12 myoblasts were seeded onto cover glasses having a diameter of 13 mm and incubated for 15, 30 and 60 minutes and induced to differentiate for 3 days. After removal from the medium, the slides were fixed with 2% paraformaldehyde in PBS for 20 min. Slides were then incubated overnight with anti-myogenin and anti-MyoD (Abcam®, USA) at dilutions of 1:100. After three washes in phosphate-buffered saline the slides were incubated with a second antibody for 60 minutes. The immunoreaction was visualized with diaminobenzidine peroxidase substrate kit (Vector Laboratories, Burlingame, CA). Negative controls were obtained by omitting the primary antibody. The cover glasses, counterstained in Mayer’s hematoxylin, were mounted and observed by light microscopy. Quantification of staining for MyoD and myogenin was performed automatically by the software ImageJ. Images were captured using an Olympus BX43 microscope. MyoD- or myogenin-positive area was calculated as the ratio of the positive area to total cell area.

### ATP evaluation

C2C12 muscle cells (1 x 10^4^ cells) were plated into 96 well plates and incubated for 24 h for cellular adhesion. After this period the venom was added (12.5μg/mL) and cells were immediately irradiated with LLL (4.6 J/cm2) in wavelengths of 685 nm and 830 nm or cells received only medium (control) and were incubated for 15, 30 and 60 minutes. ATP levels were measured using ATPlite assay (Perkin Elmer) according to manufacturer recommendations. Specifically, to measure the levels of extracellular ATP in the supernatant of cell culture, after venom incubation and LLL irradiation, the supernatant was carefully recovered (200 μL) and directly used to ATP measurement. For measurement of intracellular ATP levels, the cells were recovered and lysed using mammalian lysis cell buffer (Perkin Elmer), centrifuged and the supernatant was directly used for ATP measurement [32].

### Statistical analysis

Results were expressed as mean ± SEM. The data were compared by using ANOVA test, followed by a post-hoc test using Tukey’s multiple comparison test. The threshold of significance for the ANOVA and the Tukey’s test was fixed at p≤0.05.

## Results

### Effect of laser irradiation at 2.0, 4.6 and 7.0 J/cm^2^ on the viability of C2C12 cell exposed to venom

**Fig 1:**
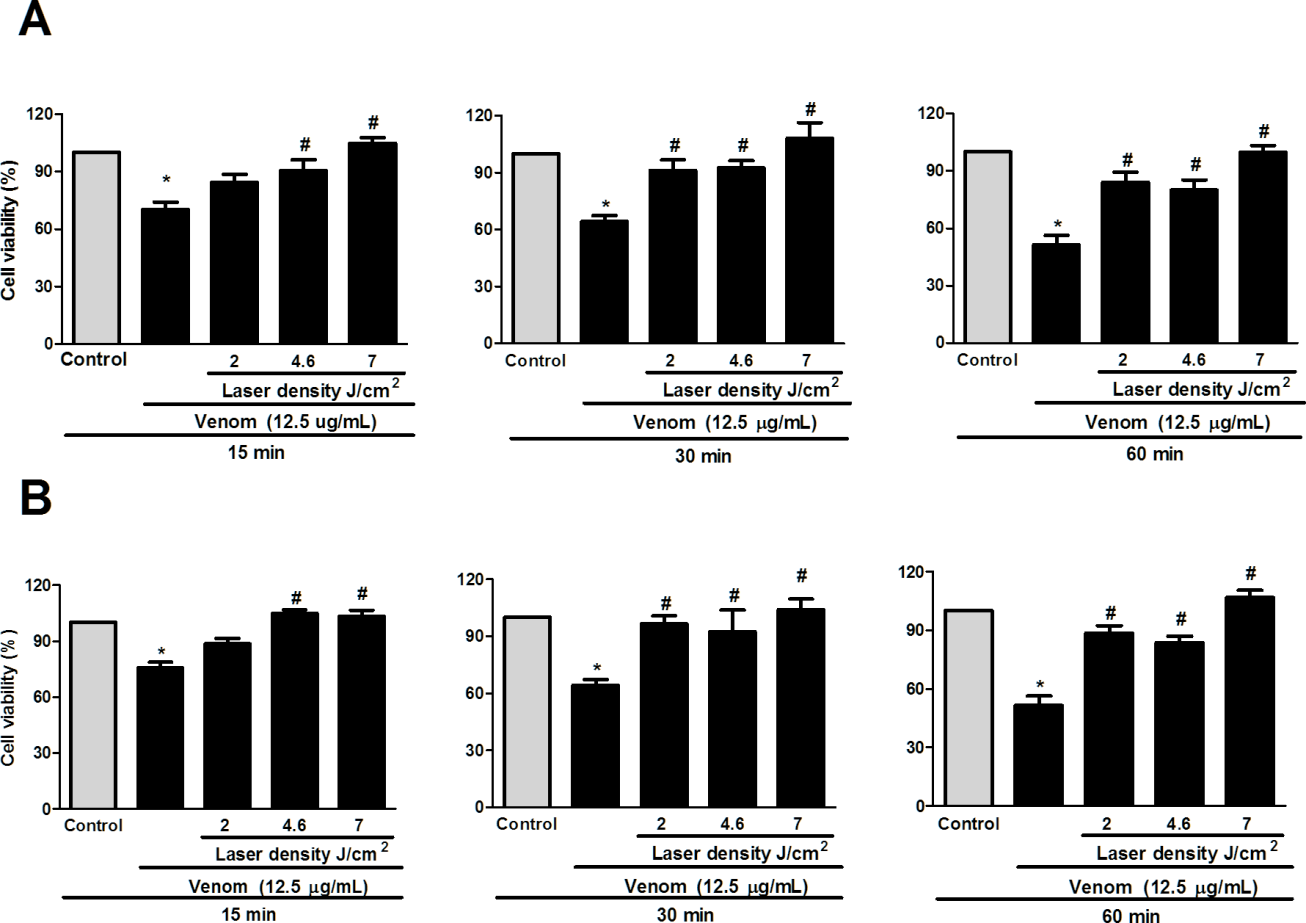
Effect of laser irradiation on the viability of C2C12 cells exposed to *B*. *jararacussu* venom. C2C12 muscle cells were plated into 96 well plates and incubated for 24 h for cellular adhesion. After this period the venom was added (12.5 μg/mL) and cells were immediately irradiated with LLL (2.0, 4.6 and 7.0 J/cm^2^) in wavelengths of (A) 685 nm and (B) 830 nm or cells received only medium (control) and were incubated for 15, 30 and 60 minutes. Cell viability was determined by MTT assay. Each value represents the mean ± SEM of three independent experiments. *p< 0.05 compared to control and **#** p< 0.05 compared to venom.

To determine the effect of various dosages of laser irradiation on the viability of C2C12 cell exposed to venom, the MTT assay was performed. Results showed that irradiation with 685 or 830 nm wavelengths caused an increase on C2C12 cell viability exposed to venom in the same manner (Fig 1). Moreover, laser irradiation increases cell viability in energy density of 4.6 and 7 J/cm^2^ at 15, 30 and 60 min when compared to cell exposed to venom. The energy density of 2 J/cm^2^ caused an increase in cell viability at 30 and 60 min of venom exposure. However, there was no statistical difference between the laser dosages. The comparison of the viability of C2C12 cells irradiated with 685 or 830 nm laser at 2, 4.6 and 7 J/cm2 energy density with that of non-irradiated control cell and in venom stimulated cells is shown in Fig 1. Based on these results, all subsequent experiments were performed with an energy density of 4.6 J/cm^2^.

### Effect of irradiated lyophilized *B*. *jararacussu* snake venom

To test if the laser irradiation was able to induce changes in the venom components, venom solution was irradiated before the incubation with the cells. Our results showed that both lyophilized irradiated and non-irradiated venom caused the same effect on the cell viability (Table 2).

**Table 2:** Effect of LLL irradiation on cell viability of C2C12 cells after exposure to irradiated *B*. *jararacussu* venom.

Cell viability (%)			
Time (min)	Venom	iVenom 685 nm	iVenom 830 nm
15	65.2 ± 3.1	68.1 ± 1.6	62.3 ± 2.9
30	52.4 ± 2.3	49.4 ± 1.6	54.3 ± 3.1
60	42.2 ± 4.3	48.1 ± 1.3	48.8 ± 1.9

C2C12 cells were plated into 96 well plates and incubated for 24 h for cellular adhesion. After this period, *B*. *jararacussu* venom (venom) or irradiated *B*. *jararacussu* venom (iVenom) was added (12.5 μg/mL) and the cells were incubated for 15, 30 and 60 min. Cell viability was determined by MTT assay.

### Effect of laser irradiation on the CK and LDH release

**Fig 2:**
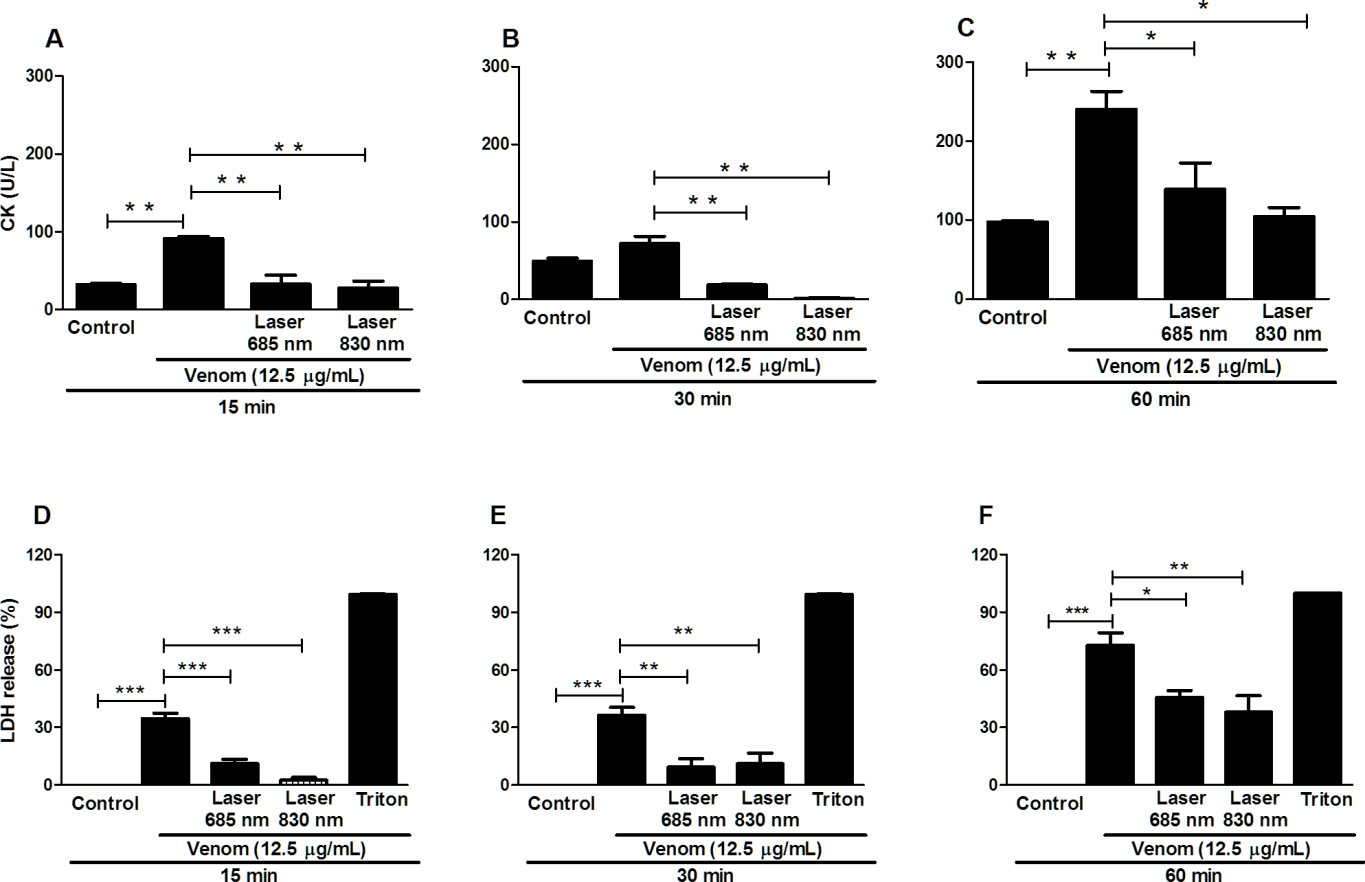
Effect of laser irradiation on CK and LDH activity of C2C12 cells exposed to *B*. *jararacussu* venom. C2C12 muscle cells were plated into 96 well plates and incubated for 24 h for cellular adhesion. After this period the venom was added (12.5μg/mL) and cells were immediately irradiated with LLL (4.6 J/cm^2^) in wavelengths of 685 nm and 830 nm or cells received only medium (control) and were incubated for 15, 30 and 60 minutes. Determination of CK activity was determined using the kit CK NAC **(A, B, C)** and LDH activity was determined using the kit LDH Liquiform **(D, E, F)**. Each value represents the mean ± SEM of three independent experiments. * p < 0.05; ** p < 0.01; *** p < 0.01 compared to venom.

To verify whether the laser protection of the myotoxic activity is related to its direct effect on muscle cells, we studied the effect of *B*. *jararacussu* venom incubation on the myoblast C2C12 cell line on the basis of CK activity and LDH release. The results showed a reduction in the CK levels in the groups treated with both laser (685 nm and 830 nm) in all periods analyzed (Fig 2A, 2B and 2C), when compared to the group that received only venom. Likewise, cells treated with venom presented increased levels of LDH activity in their supernatant compared with cell that received only medium. Laser irradiation caused an inhibition of the LDH release, in all period studied, and by both wavelengths (Fig 2D, 2E and 2F).

### Effect of laser irradiation on differentiation of C2C12 myoblast

**Fig 3:**
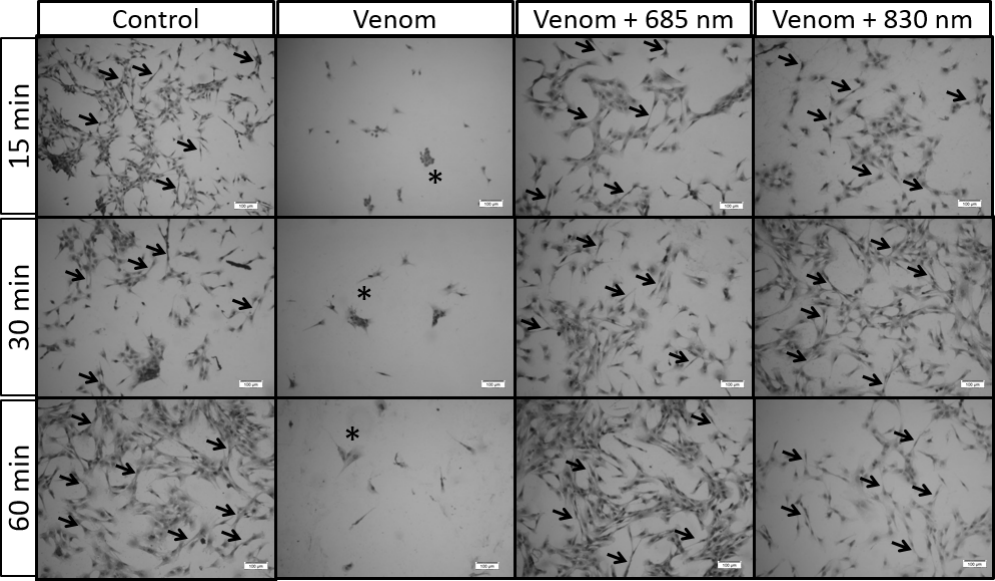
Effect of laser irradiation on differentiation of C2C12 cells exposed to *B*. *jararacussu* venom. Light micrographs of C2C12 cells treated with venom and laser irradiation (as described in material and methods). Control cells incubated with 2% horse serum shows normal appearance with elongated and thin shape, characteristic of myoblast differentiation (arrows). There was no viable cell in the group incubated with *B*. *jararacussu* venom (*). Note that in the group of cell incubated with venom and irradiated with both laser wavelengths present morphology similar to control cells in differentiation process (arrows). The morphology of cells was observed under an optical microscope. Hematoxylin & eosin staining. Increased 10X.

The results showed that 4 days of differentiation induction, control cells incubated with 2% horse serum showed an elongate and thin form and fused with each other, which characterizes the differentiation of C2C12 myoblast cell (arrows). After 4 days of differentiation induction, there were no viable cells (*) in the group that was incubated with venom, this occurred in all periods of venom incubation (15, 30 or 60 min). However, cells incubated with the venom and treated with both laser showed the same cell morphology of control cells (arrows) (Fig 3).

### Effect of laser irradiation on Myogenin and MyoD protein expression

**Fig 4:**
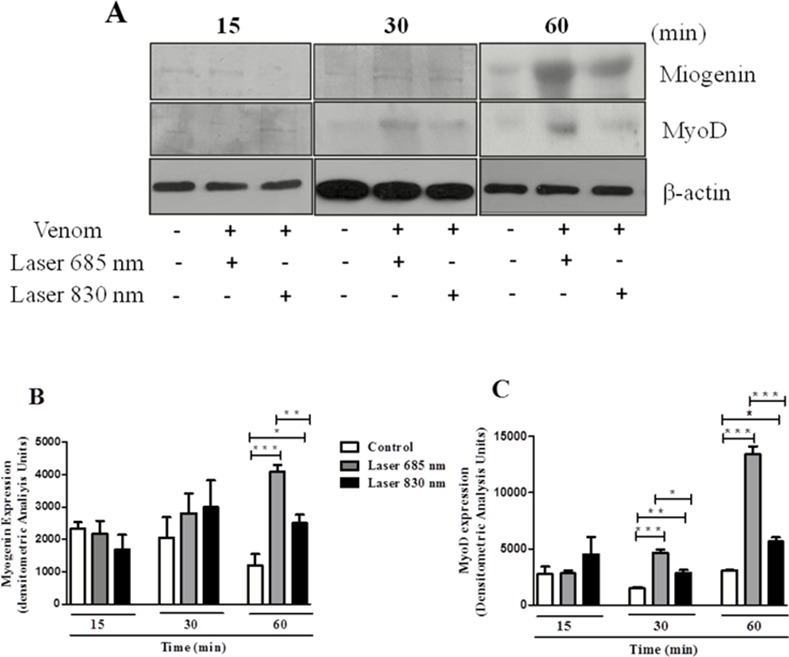
Effect of laser irradiation on the expression of MyoD and myogenin protein by C2C12 cells exposed to *B*. *jararacussu* venom. The cells were collected 3 days after the venom incubation for 15, 30 or 60 min and treated with laser irradiation or culture medium (control). **(A)** Representative blot from three independent experiments. Bar graph showing densitometric analysis of immunoreactive myogenin **(B)** and MyoD **(C)** band intensities normalized to β-actin. Results are expressed as mean ± S.E.M. from three experiments * p < 0,05, ** p< 0,01 e *** p<0,01.

Expression of MyoD and myogenin protein was analyzed in C2C12 cells collected 3 days after incubation with the venom and treatment with laser. Cells that were incubated with venom for 15 min and treated with laser, showed no statistical difference in the expression of these proteins compared to control group (Fig 4A). After 30 minutes, there was a significant increase in MyoD protein expression but not myogenin protein by two wavelengths studied (Fig 4B) and within 60 min incubation with the venom and treatment with both laser wavelengths caused a significant increase of MyoD and myogenin protein expression in C2C12 cells (Fig 4C). In addition, it can be observed that the laser at the wavelength 685 nm showed a more intense expression of both analyzed protein compared to the wavelength of 830 nm in all the periods of time where an increase in expression of these proteins occurred (Fig 4D and 4E).

**Fig 5:**
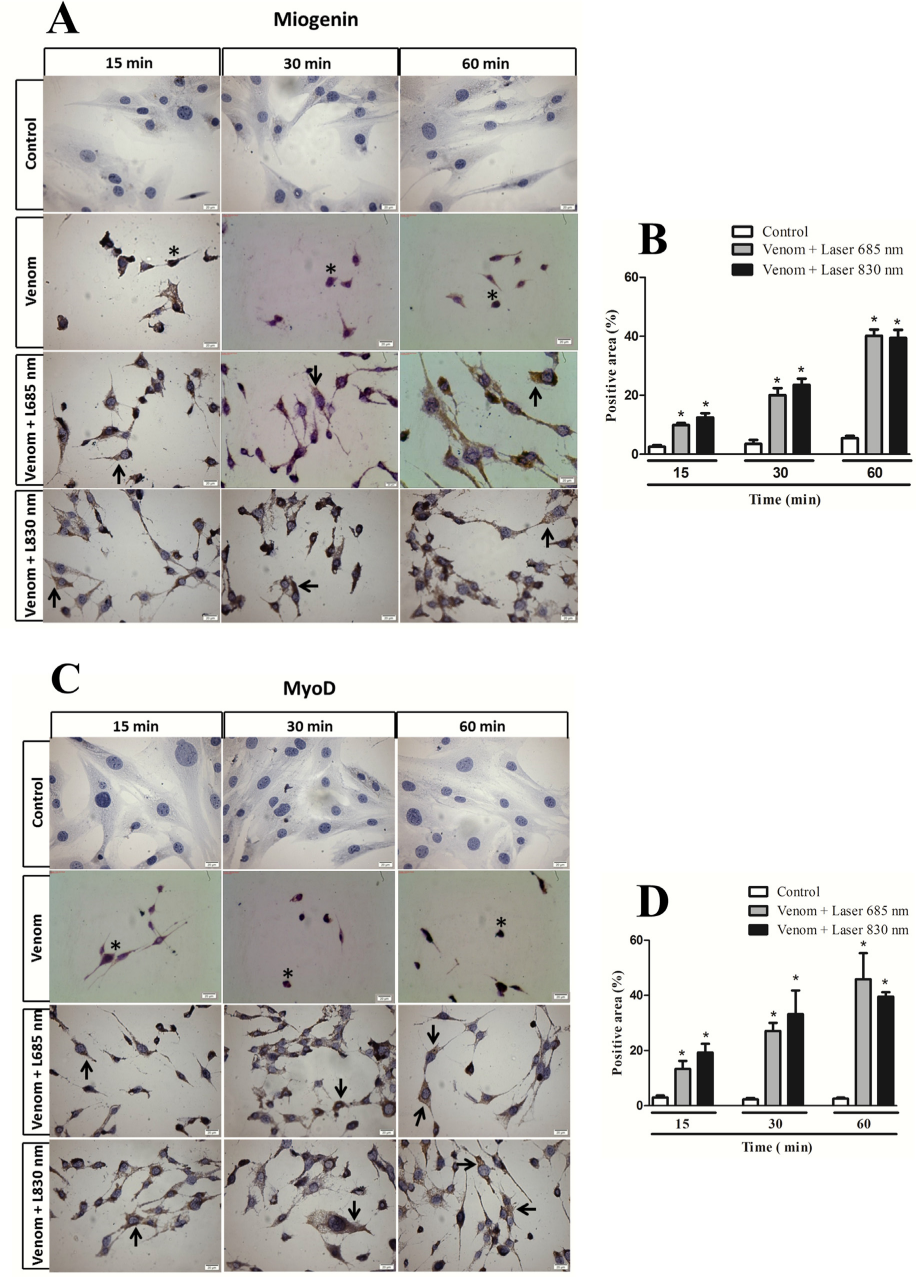
Immunohistochemical analysis for MyoD and myogenin expression of cells exposed to *B*. *jararacussu* venom and laser irradiated. The cells were collected 3 days after the venom incubation for 15, 30 or 60 min and treated with laser irradiation or culture medium (control). Representative images of immunohistochemical analysis of myogenin **(A)** and MyoD **(C).** A positive myogenin and MyoD immunoexpression was detected by 685 and 830 nm wavelength in all period analyzed (arrows). Bar, 20 μm. Graphs show the immunohistochemical quantifications carried out in 20x images of myogenin (B) and MyoD (D). Results are expressed as mean ± S.E.M. from three experiments * p< 0.05 vs. its own control.

Immunohistochemistry data for myogenin and MyoD are summarized in Fig 5. In the control group, a weak immunoexpression of myogenin or MyoD was detected (Fig 5A and 5C, control). Nevertheless, in the venom group exposed to LLL irradiation at 4.6 J/cm^2^, a positive myogenin and MyoD immunoexpression was detected by 685 and 830 nm wavelength (Fig 5A and 5C) in all period analyzed (arrows). Quantification of immunostaining showed a significantly higher myogenin and MyoD in irradiated cells compared to control cells (Fig 5B and 5D). Regarding cell treated with venom alone, just as it occurred in differentiation experiments, after 3 days of differentiation induction, there were no viable cells (*) this occurred in all periods of venom incubation.

### Effect of laser irradiation on ATP levels

**Fig 6:**
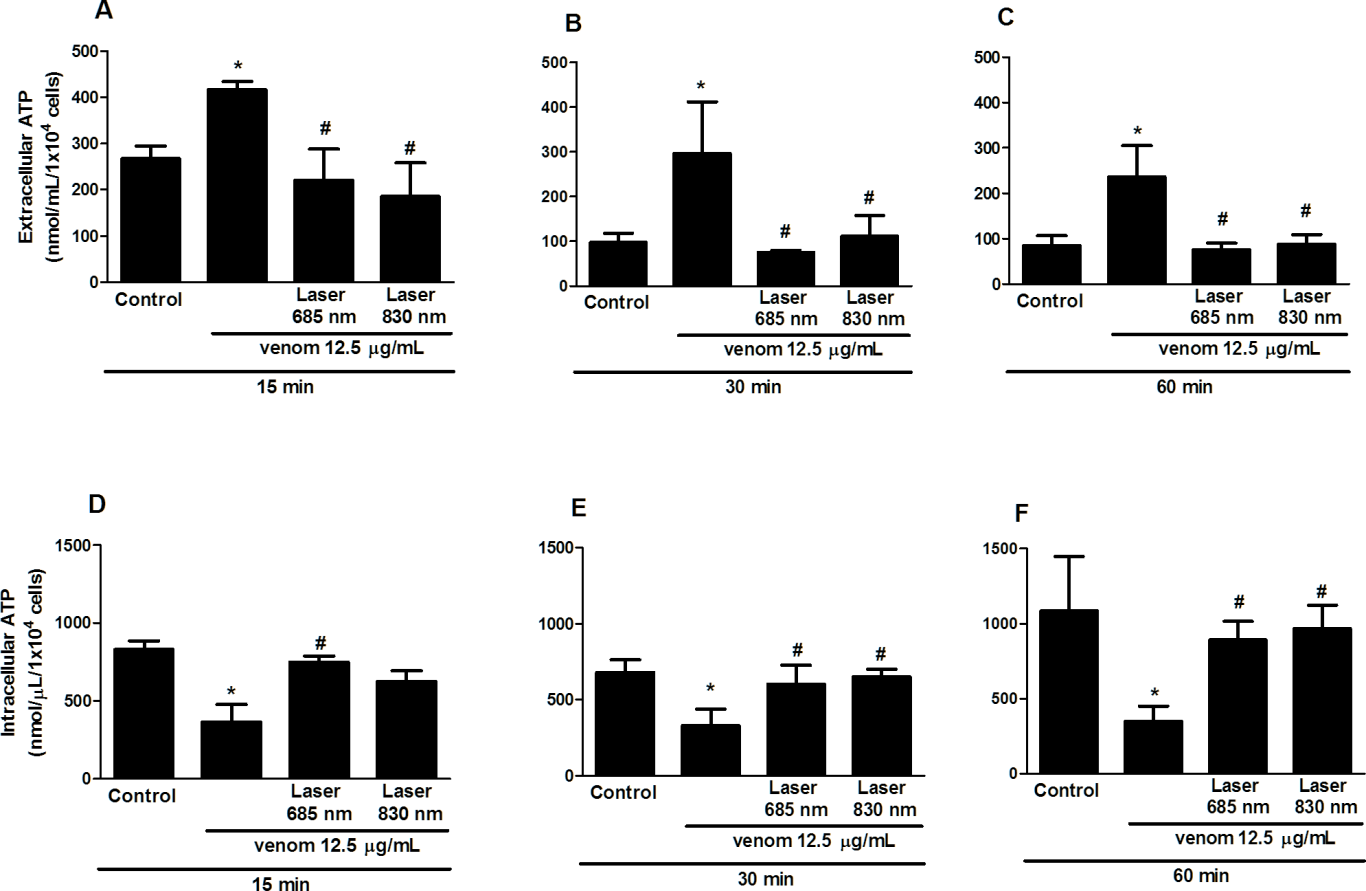
Effect of laser irradiation on ATP levels of C2C12 cells exposed to *B*. *jararacussu* venom. C2C12 muscle cells were plated into 96 well plates and incubated for 24 h for cellular adhesion. After this period the venom was added (12.5 μg/mL) and cells were immediately irradiated with LLL (4.6 J/cm^2^) in wavelengths of 685 nm and 830 nm or cells received only medium (control) and were incubated for 15, 30 and 60 minutes. Determination of ATP levels was assessed using the ATPlite assay (Perkin Elmer). Each value represents the mean ± SEM of three independent experiments. *p < 0.05 compared to control; #p < 0.05 compared to venom.

To further evaluate the possible mechanism involved in the cytoprotection observed in our experimental model, intra and extracellular levels of ATP was measured. Results showed that cells exposed to venom demonstrated a reduction in the intracellular levels of ATP while was observed an ATP accumulation in the intracellular milieu, in all period analyzed (Fig 6). Irradiation with red laser showed a return to control levels of both intracellular and extracellular ATP levels, at 15, 30 and 60 min of venom incubation and laser irradiation (Fig 6). The infrared laser demonstrated the same effect seen with the red laser in the intra- or extracellular level, with the exception in intracellular ATP level at 15 min that showed no statistical difference compared to venom group (Fig 6D).

## Discussion

In the present study, the myoblast C2C12 cell line was used to examine the direct effect of *B*. *jararacussu* venom on muscle cell. The use of skeletal muscle myoblasts/myotubes as targets for snake venom/toxins has been suggested as a viable *in vitro* model to study myotoxic mechanisms, as it correlates with *in vivo* myotoxicity [33]. Our results have demonstrated a cytotoxic effect of *B*. *jararacussu* snake crude venom on a cultured myoblast cell line. These results are in agreement with cytotoxic activities from crude venom and venom components, such as myotoxins and metalloproteinases, studied on C2C12 cell [29, 33–36]. In order to further characterize *B*. *jararacussu* venom cytotoxicity, we examined plasma membrane integrity by LDH and CK release in the supernatant. Both test showed a large increase in the release of these enzymes compared to control cells, confirming the venom cytotoxicity on myoblast cells.

In spite of the fact that many studies of photobiomodulation therapy on the local reaction caused by Bothrops venoms have demonstrated its beneficial effects *in vivo* [18, 22, 23] little is known about how laser treatment is able to affect cellular systems involved in bothropic venom-induced local effects, particularly in myonecrosis, and what are the molecular mechanisms involved in this process. In this study, the effect of laser on the cytotoxicity caused by *B*. *jararacussu* venom was evaluated using the laser density of 2.0, 4.6 and 7,0 J/cm^2^ in two wavelengths; a red wavelength at 685 nm and an infrared wavelength at 830 nm, applied to the monolayer of cells immediately after the venom incubation. The laser energy densities were chosen based on the literature that shows, in cultured cells, a beneficial effect of red or infrared laser as low as 3 or 5 J/cm^2^ and a larger dose, over 16 J/cm^2^ lose the beneficial effect and may even become harmful [30]. The results obtained in our study have shown that laser irradiation in a dose of 2.0, 4.6 and 7.0 J/cm^2^ reduced considerably the cytotoxicity caused by *B*. *jararacussu* venom in myoblast culture. In agreement with our study, Dourado et al. [22] and Barbosa et al. [18] in an *in vivo* study found that the laser in wavelength at 904 and 685 nm and energy density of 4 J/cm^2^ and 4.2 J/cm^2^, respectively, was able to significantly decrease venom-induced myonecrosis demonstrated by histology and confirming by reduced levels of CK release. Based on these findings, we selected the energy density of 4.6 J/cm^2^ for all subsequent studies.

In order to verify whether the laser irradiation would be capable of altering the venom components and thus decrease its cytotoxicity, the lyophilized venom solution was irradiated before incubation with cells. Results demonstrated that irradiate *B*. *jararacussu* venom showed the same cytotoxicity observed with non-irradiated venom. These results are in agreement with studies using an *in vivo* model showing that the irradiated venom induced the same level of muscle edema [19] and neuromuscular blockade [21] as the non-irradiated venom. These results indicate that the laser irradiation does not modify the venom components but acts in the cellular response.

The main venom component that causes muscle cell damage is PLA_2_ myotoxin [37]. It has been demonstrated that myotoxic PLA_2_s target the sarcolemma and induce an acute degeneration of skeletal muscle fibers [38]. However, the mechanism of myotoxicity exerted by venom PLA_2_s are still only partially understood [39]. Gutierrez and Ownby (2003) [37] proposed two main types of damage induced by myotoxic PLA_2_s on plasma membrane of muscle cells: (1) a perturbation in the integrity of the bilayer by a mechanism independent of phospholipid hydrolysis, and (2) a membrane disruption based on enzymatic phospholipid degradation. Furthermore, Cintra-Fransichineli et al. (2010) [6] showed that myotoxins isolated by Bothrops venom promote a fast efflux of K^+^ and ATP into the extracellular environment and reveals an indirect mechanism of ATP-mediated mechanism through which muscle are affected. It is possible that, in our experimental model, the laser is protecting the myoblast by a mechanism independent of enzymatic phospholipid hydrolysis, this hypothesis is based on the fact that low level light is absorbed by components of the respiratory chain which leads to changes in both mitochondria and the cytoplasm initiating an intracellular signaling cascade that promotes cellular cytoprotection [16, 17]. To test this hypothesis, we measured the amount of intra- and extracellular ATP levels. Our results showed that the laser irradiation has prevented the ATP release into the extracellular and increased its intracellular levels. These results provide the first evidence that LLL irradiation can modulate ATP synthesis after venom exposure. It has been demonstrated that isolated myotoxins from *B*. *asper* venom induced a release of large amount of K+ and ATP from skeletal muscle. They also show that ATP released amplifies the effect of the myotoxins, which spreads and causes much larger damage than that directly caused by the myotoxins [6]. We hypothesize that the biomodulatory effect caused by LLL irradiation on ATP levels could initiate a signaling cascade that promotes cellular cytoprotection observed in our study. Furthermore, the decrease in extracellular ATP release seen after LLL irradiation could also magnify the effects of irradiation to reduce the harmful local effects caused by bothropic venom observed in *in vivo* studies, such as inflammation [19], hyperalgesia [20] and hemorrhage [23].

After muscle injury, satellite cells become activated, divide and differentiate which leads to the formation of new myofibers or to the repair of existing fibers [40]. With the observation that laser protects the C2C12 cells against *B*. *jararacussu* venom, we further evaluate whether these cells would be able to differentiate into myotubes after the venom exposure and laser irradiation. Results demonstrated that, besides the cytoprotection caused by laser irradiation in two wavelengths studied, these cells were also able to differentiate, verified by elongated cells featuring fusion, consistent with the typical morphology of differentiation in muscle cells [41]. It is noteworthy that after four days, there were no viable cells in the group that was exposed to venom and did not receive the laser irradiation, confirming the cytoprotective effect caused by photobiomodulation. The regulation process of forming new muscles involves the appropriate activation, proliferation and differentiation of myogenic cells and depends on the expression and activity of transcription factors known as myogenic regulatory factors [42]. Studies have shown that MyoD and myogenin myogenic regulatory factors are considered marker muscle growth as they can regulate the division of satellite cells [43]. In addition, MyoD and myogenin regulate the expression of genes necessary for terminal differentiation, such as the myosin heavy chain gene family [43]. In our experimental model the differentiation of myotubes observed by histology after the laser irradiation was accompanied by an increase of MyoD and myogenin myogenic regulatory factors. In this study, an important finding is that the expression of both MyoD and myogenin was increased by LLL irradiation, which could contribute in the promotion of regenerating muscle fibers. These findings corroborate those of Rodrigues et al. [44] and Brunelli et al. [45], which demonstrated that treatment with low level laser caused an increase in myogenin and MyoD gene expression during healing muscle in a rat model of muscle cryolesion. Herein, the present results suggest that laser irradiation was able to modulate the expression of MyoD and myogenin by C2C12 cells, and it appears likely that this modulation plays a role in the muscle protection observed after bothropic venom in response to photobiomodulation.

In conclusion, this study reveals that photobiomodulation with low level laser caused a protective effect on C2C12 myoblasts after *B*. *jararacussu* venom incubation involving intra and extracellular ATP modulation. Moreover, laser irradiation was able to promote differentiation of C2C12 cells through up regulation of myogenic factors, MyoD and myogenin. Although there are still many questions to be answered about the mechanisms by which laser irradiation causes protective effects on venom-induce myonecrosis, the current investigation demonstrated that the use of photobiomodulation could be an effective therapeutic tool associated with the existing antivenom therapy in the management of snakebites. However, there is a need for further *in vitro* studies to improve knowledge about the mechanisms involved in laser effect on local damage caused by bothropic venoms.
